# Theoretical modelling of epigenetically modified DNA sequences

**DOI:** 10.12688/f1000research.6148.2

**Published:** 2015-05-06

**Authors:** Alexandra Teresa Pires Carvalho, Maria Leonor Gouveia, Charan Raju Kanna, Sebastian K. T. S. Wärmländer, Jamie Platts, Shina Caroline Lynn Kamerlin

**Affiliations:** 1 Science for Life Laboratory, Department of Cell and Molecular Biology, Uppsala University, Uppsala, S-751 24, Sweden; 2 Department of Immunology, Genetics and Pathology, Rudbeck Laboratory, Uppsala, S-751 85, Sweden; 3 Department of Biochemistry and Biophysics, Stockholm University, Stockholm, S-106 91, Sweden; 4 School of Chemistry, Cardiff University, Cardiff, CF10 3AT, UK

**Keywords:** Epigenetics, DNA modifications, DNA methylation, Density functional theory, hybrid QM/MM calculations, DNA model systems

## Abstract

We report herein a set of calculations designed to examine the effects of epigenetic modifications on the structure of DNA. The incorporation of methyl, hydroxymethyl, formyl and carboxy substituents at the 5-position of cytosine is shown to hardly affect the geometry of CG base pairs, but to result in rather larger changes to hydrogen-bond and stacking binding energies, as predicted by dispersion-corrected density functional theory (DFT) methods. The same modifications within double-stranded GCG and ACA trimers exhibit rather larger structural effects, when including the sugar-phosphate backbone as well as sodium counterions and implicit aqueous solvation. In particular, changes are observed in the buckle and propeller angles within base pairs and the slide and roll values of base pair steps, but these leave the overall helical shape of DNA essentially intact. The structures so obtained are useful as a benchmark of faster methods, including molecular mechanics (MM) and hybrid quantum mechanics/molecular mechanics (QM/MM) methods. We show that previously developed MM parameters satisfactorily reproduce the trimer structures, as do QM/MM calculations which treat bases with dispersion-corrected DFT and the sugar-phosphate backbone with AMBER. The latter are improved by inclusion of all six bases in the QM region, since a truncated model including only the central CG base pair in the QM region is considerably further from the DFT structure. This QM/MM method is then applied to a set of double-stranded DNA heptamers derived from a recent X-ray crystallographic study, whose size puts a DFT study beyond our current computational resources. These data show that still larger structural changes are observed than in base pairs or trimers, leading us to conclude that it is important to model epigenetic modifications within realistic molecular contexts.

## Introduction

The standard four-letter alphabet used to encode genetic information in DNA is a central tenet of molecular biology. However, *in vivo* chemical modification of bases can expand this alphabet markedly, giving rise to a host of important biological phenomena ^1^. Epigenetic modifications, most importantly DNA methylation and histone variation, have the potential to affect gene expression, and are believed to play a major role in the complex pattern of development and differentiation of multi-cellular organisms. Fascinatingly, such modifications may be heritable despite not affecting DNA sequence, although the mechanism(s) by which this could be achieved are currently unknown. For details of currently proposed mechanisms please see reference 1 and work cited therein.

**Figure 1.**
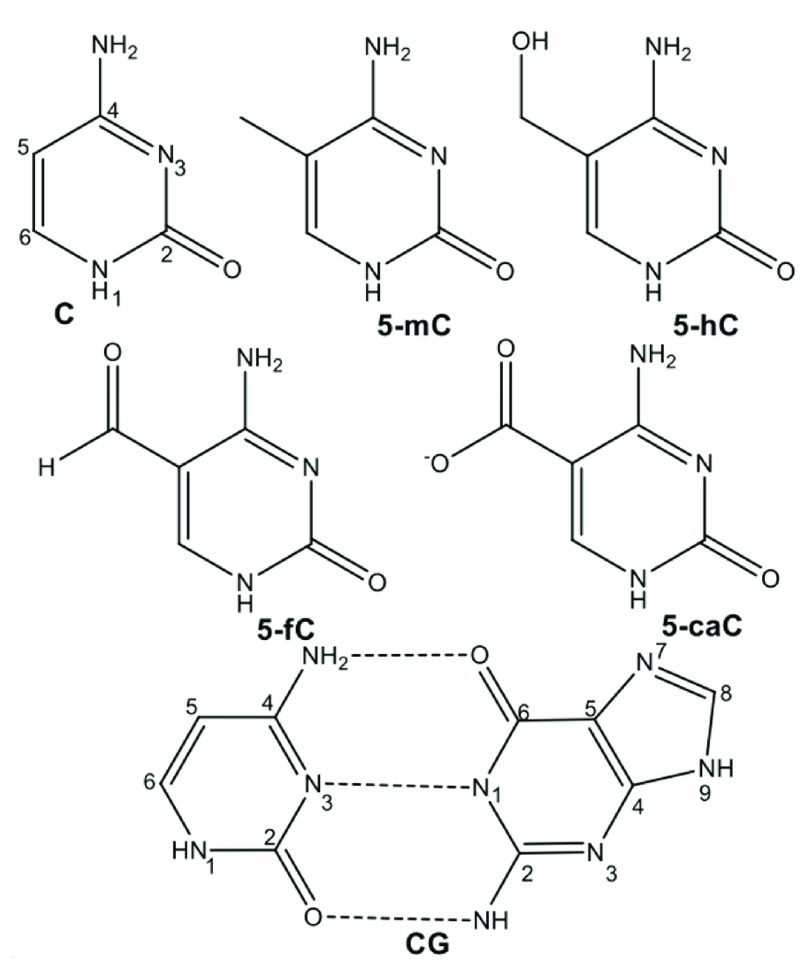
Structures of cytosine and its epigenetic modifications.

The most common and biologically important such modification involves methylation of the 5 position of cytosine (C) to form 5-methylcytosine (5-mC), illustrated in Figure 1. This does not strongly affect the ability of the base to pair with guanine (G), and in mammals is generally found in CpG sequences, though bacteria and plants display less sequence specificity ^2^. Oxidation of 5-mC can form 5-hydroxymethylcytosine (5-hmC), which is believed to be involved in regeneration of C *via* ten-eleven translocation (TET) proteins. Moreover, recent work has shown that 5-formylcytosine (5-fC), and 5-carboxycytosine (5-caC) are present in stem cells and organs of mice ^3^.

The structural consequences of cytosine methylation and related modifications were the focus of a recent study ^4^ that used X-ray crystallography to show that incorporation of 5-mC or 5-hmC at different points in the d(CGCGAATTCGCG) dodecamer has a negligible effect on both local (base pair) and global (helical) geometry, although specific preference for the orientation of the hydroxyl group in the latter was clearly evident. However, while elegant, the resolution of these studies (between 1.42 and 1.99 Å) may mean that subtle structural changes could go unnoticed. Therefore, molecular modelling, whether based on quantum or classical mechanics, has the potential to contribute significantly in this field. Quantum mechanical models, typically using density functional theory (DFT), have been used to examine the base pairing and stacking of both unmodified (wild-type) and 5-mC DNA. Many groups, including those of Fonseca-Guerra ^5– 7^, Šponer ^8– 13^, Leszczynski ^14– 16^ and others have used DFT to great effect in understanding the structure and properties of unmodified DNA. Regarding epigenetic modifications in particular, Acosta-Silva *et al.* ^17^ showed in this manner that methylation enhances stacking interactions, and can produce local distortions in base-pair step parameters, most notably in the slide parameter. Yusufaly *et al.* used similar calculations to show that methylation can induce over-twisting as well as softer modes for distortion from the global energy minimum ^18^. We recently employed classical mechanics to examine not only the structure but also the flexibility of different DNA sequences with methyl and hydroxymethyl substituents ^19^. Through use of extended molecular dynamics (MD) simulations, we showed that structural effects are subtle, but that epigenetic modifications can give rise to changes in twist, roll and tilt angles that are markedly sequence-dependent. Moreover, introduction of 5-mC within a sequence that already contains hydrophobic groups in the major groove strongly affects hydration patterns, whereas an isolated 5-mC has a lesser effect on solvation and structure.

In this work, we use DFT and QM/MM methods to examine model systems containing modified cytosines. These range from individual base pairs, through double-stranded trimers, to heptamers. By including the sugar-phosphate backbone, sodium counterions and solvent we suggest that these are more realistic models than previous work using similar methods. However, a trimer of DNA brings us close to the size limit for application of DFT with the computing resources available to us. We therefore test and employ hybrid QM/MM methods for larger systems, in which the central bases are treated with dispersion-corrected DFT, while outer bases, sugar-phosphate backbone and solvent (where appropriate) with a molecular mechanics approach, thus allowing accurate and efficient description of systems consisting of hundreds of atoms.

## Computational methodology

The initial structures of model systems were built in the canonical B-DNA geometry, using the w3DNA server ^20^. Hydrogen atoms were added to the system according to expected protonation states at physiological pH using the Molecular Operating Environment (MOE) software package, and Na ^+^ were added manually in the vicinity of each phosphate group to produce an overall neutral structure. Where relevant, the central cytosine was also manually modified, and the results of all simulations were analysed using the X3DNA software package ^21, 22^. Atomic coordinates of wild-type, methylated and hydroxymethylated DNA dodecamers were obtained from X-ray structures deposited in the Protein Data Bank (PDB IDs: 1BNA, 4GJU, 4GLG, 4GLH and 4GLC) ^23^, and truncated to 5´-ATTCGCG-3´ heptamers containing a single modification on the central C. All DNA termini were capped with methyl groups for simplicity.

All DFT calculations were performed with the Gaussian09 simulation package ^20^, using default SCF and geometry optimisation criteria. Throughout, we use Grimme’s B97-D functional ^24^, that includes an explicit correction for the missing dispersion term in conventional DFT functionals, with either def2-TZVP or 6-31+G(d,p) basis set. This was previously recommended after thorough benchmarking for thermochemistry, kinetics, and non-covalent interactions ^25^. All such calculations took advantage of the density fitting approximation, and where appropriate included the effect of aqueous solvation *via* the use of the polarized continuum model (PCM) ^26^. Binding energies are corrected for the effects of basis set superposition error using the counterpoise method ^27^.

Hybrid QM/MM calculations were performed using the ONIOM approach with electrostatic embedding ^28^, as implemented in Gaussian09. The boundary between the quantum and classical regions was chosen as the N-C1’ glycosidic bond in the relevant nucleotide. The QM regions were saturated by the use of a “link” hydrogen atom placed along the N-C1’ vector at an idealized distance, and were modelled at the B97-D/6-31+G(d,p) level of theory, again within PCM water. The MM part of these calculations employed the AMBER force field parm96 ^29^, as defined within Gaussian09. The subtractive nature of the ONIOM method means that undefined terms in the MM expression do not contribute to the overall energy if the relevant atoms are entirely within the QM region, making it ideally suited for the purposes of the current study. We note that this approach has been widely adopted for QM/MM studies of DNA and related structures ^30– 32^. Pure molecular mechanics (MM) geometry optimisation was also performed using the GROMACS simulation package ^33^ and the AMBERParmbsc0 force field ^34^, including RESP charges derived for modified bases in our previous work ^19^, in explicit aqueous phase, specifically TIP3P water ^35^ with Na ^+^ and Cl ^-^ counter ions to create a neutral system. Full details of the MM treatment can be found in ref. 19.

## Results and discussion

### Gas-phase base pairs

To examine the effect of modifications on base pairing we examined the structure and energy of gas-phase CG pairs in both hydrogen bonded and stacked orientations, with results reported in Table 1 and Table 2 respectively. These data show that methylation has little effect on the geometry or stability of the Watson-Crick base pair. The presence of a hydroxymethyl slightly weakens the N _4_-H _4_…O _6_ H-bond, perhaps due to the proximity of CH _2_OH and NH _2_ groups, reported as X…H _4_ in Table 1. Formyl has a larger effect overall, lengthening N _3_…H _1_-N _1_ and O _2_…H _2_-N _2_ H-bonds and hence reducing binding by over 3 kcal/mol, presumably due to the electron withdrawing effect of the formyl group, an effect that has been clearly documented before ^36– 38^. The pattern of changes induced by carboxylate is different from all other modifications, lengthening the peripheral H-bonds N _4_-H _4_…O _6_ and O _2_…H _2_-N _2_ markedly, but shortening N _3_…H _1_-N _1_. Despite this weakening, the carboxylate-substituted cytosine binds most strongly to guanine, presumably due to ion-dipole interactions within the anionic system. Both formyl and carboxylate contain close O…H _4_ contacts, but overall the proximity of these groups does not appear to be related to strength or geometry of binding.

**Table 1.** Hydrogen bond lengths and binding energies of CG Watson-Crick base pairs from B97-D/def2-TZVP (Å and kcal/mol).

	N _4_-H _4_…O _6_	N _3_…H _1_-N _1_	O _2_…H _2_-N _2_	X…H _4_^a^	Binding Energy
C	1.663	1.819	1.835	2.455	-31.19
5-mC	1.660	1.817	1.822	2.375	-31.70
5-hC	1.689	1.822	1.835	2.145	-28.63
5-fC	1.670	1.834	1.884	1.990	-28.07
5-caC	1.698	1.778	1.874	1.674	-34.62

^a^ X refers to the atom of the substituent on position 5 closest to H _4_.

**Table 2.** Geometry and binding energies of stacked CG base pairs from B97-D/def2-TZVP (Å, ° and kcal/mol).

	Cent… Cent ^a^	Dihedral ^a^	Binding Energy
C	3.381	9.0	-16.07
5-mC	3.310	12.5	-17.52
5-hC	3.361	9.4	-22.12
5-fC	3.451	2.9	-14.65
5-caC	3.823	32.6	-15.56

^a^ Cent…Cent refers to the distance between centroids of 6-membered rings; Dihedral refers to the angle between mean planes of rings.

As well as the effect on H-bonding, epigenetic modifications can alter the stacking behaviour of DNA bases. Table 2 reports geometrical details, as well as binding energies, of the five modified cytosines considered here stacked with guanine. All such calculations started from the idealised B-DNA orientation (Cent…Cent = 4.390 Å, Dihedral = 4.9°), and overall this is retained in our gas-phase DFT optimisation. Table 2 shows that methylation leads to closer contact and greater stabilisation between bases, as might be expected due to the increased polarizability of this modified base. Hydroxymethylation leads to the most stable pair considered here, largely due to a strong H-bond between the H—O of hydroxymethyl and O6 of guanine (H…O = 1.770 Å), whereas formylation leads to longer, weaker interaction between bases. Carboxylate-substituted cytosine is the only case considered here that loses the approximately parallel orientation of bases. This appears to be driven as much by repulsion between the carboxylate group and C=O _6_ of guanine as by H-bonding.

### Double-stranded DNA trimers

While these gas-phase dimers give useful information on the intrinsic effect of modifications on cytosine’s ability to interact with guanine, environmental effects including the DNA sequence, sugar-phosphate backbone and solvent will play a major role in determining their effect in real systems. In order to better simulate the behaviour of modified cytosines in real systems, structures of double-stranded d(GCG) and d(ACA), as well as epigenetic modifications to the central cytosine were optimised using DFT in continuum solvent (PCM), and the resulting geometries of the local base pairs were analysed in the coordinate frame recommended by Olson *et al.* ^39^. Unlike the free dimers considered above, modifications have only subtle effects on this larger structure, which retains the overall canonical B-DNA shape of the unmodified WT structure.

**Figure 2.**
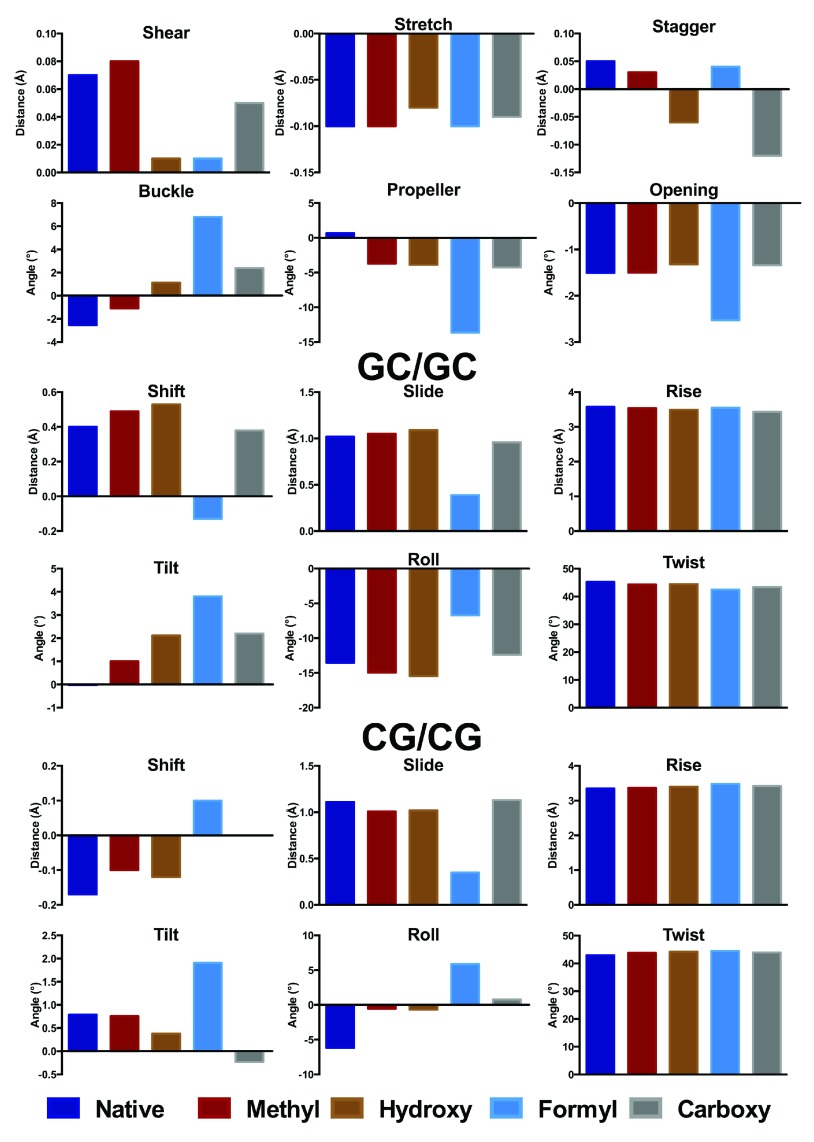
Base pair (A) and step (B, C) parameters for central GC and modifications thereof in d(GCG) (Å and °). The corresponding data are provided in Data file 1.

**Figure 3.**
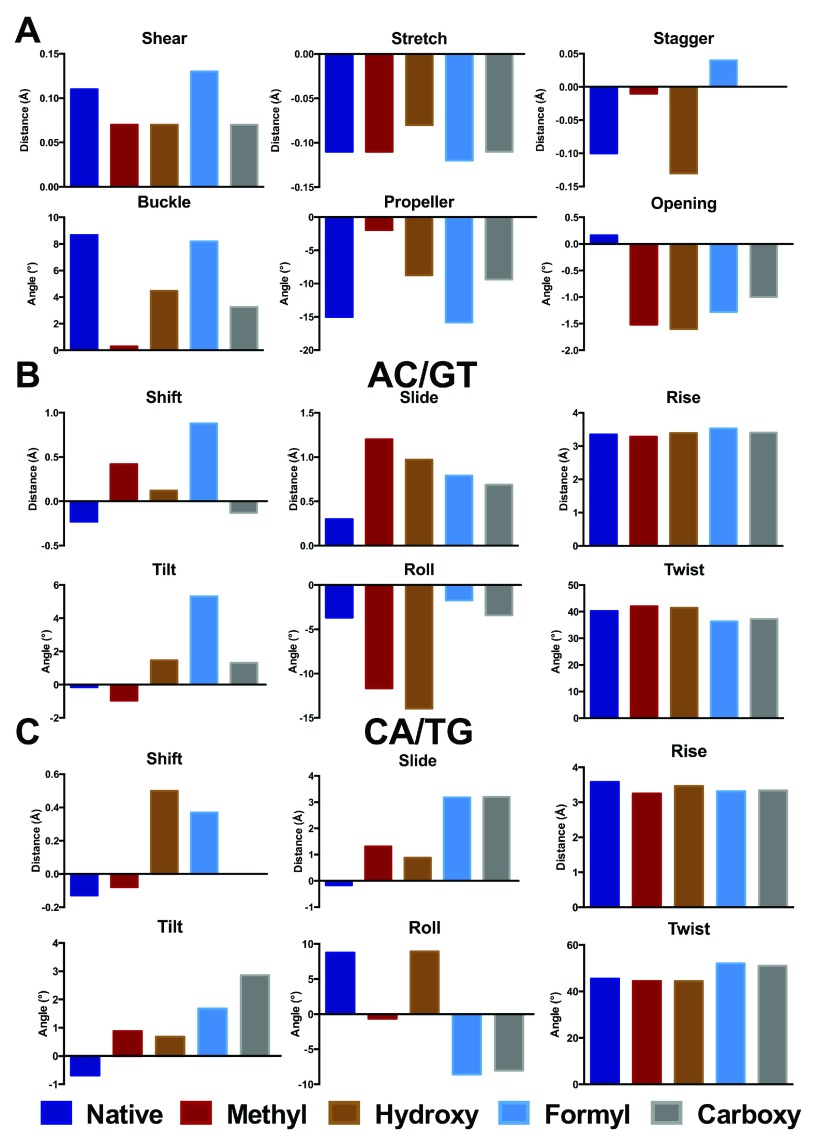
Base pair (A) and step (B, C) parameters for central GC and modifications thereof in d(ACA) (Å and °). The corresponding data are provided in Data file 1.

Following Zubatiuk *et al*. ^14^, we summarise key aspects of trimer structure, which are displayed graphically in Figure 2 and Figure 3. The corresponding values are tabulated in Data file 1 of the Supporting Information, with the base step and local helical parameters tabulated in Data file 2. As with Zubatiuk *et al*. ^14^, base pair step parameters are averaged over 3´ and 5´ directions. In the GCG oligomer, methylation has only a small effect on base pair distances, but does alter the propeller angle by over 4°. Hydroxymethylation has a larger effect on the GCG oligomer, especially on the stagger, buckle and propeller, whereas the stretch and opening parameters are much less affected. Formyl does not strongly affect base pair distances but does change angles substantially, especially buckle and propeller, which change by as much as 10°. In contrast, carboxylate induces a large change in stagger but only small changes in angular geometry. Base pair step parameters for d(GCG) in general are less affected than those for the base pair noted above, with the exception of formyl which exhibits smaller slide and less negative roll values than unmodified DNA.

Rather larger changes are evident on modification of d(ACA), as shown in Figure 3. In this case, even methylation induces significant changes in distances, especially stagger which increases by 0.1 Å, and angles (buckle and propeller change by 8 and 13°, respectively). At the base pair step level, methylation gives rise to substantial increase (0.9 and 1.5 Å) in slide and more negative roll in both 3´ and 5´ directions. Less apparent in Figure 3, but still notable, are changes in rise that are 0.1 and 0.3 Å smaller in the methylated structure, reflecting the greater stacking that results from addition of a methyl group. Other modifications induce different patterns of structural change: for the central base pair these changes are typically smaller than for methylation, but for base pair steps much larger changes are found in some parameters. Most notable of these are slide, which changes by over 3 Å and roll (up to 17°) in the 3´ direction, in a similar way to that reported previously for smaller systems ^17, 18^. Other parameters such as the width of the DNA strand, measured as the distance between C1´ nuclei, and virtual angles λ _Y_ and λ _R_, which describe the pivoting of complementary bases in the base-pair plane, vary only slightly from the idealised values for B-DNA.

### QM/MM studies of double-stranded oligomers

**Figure 4.**
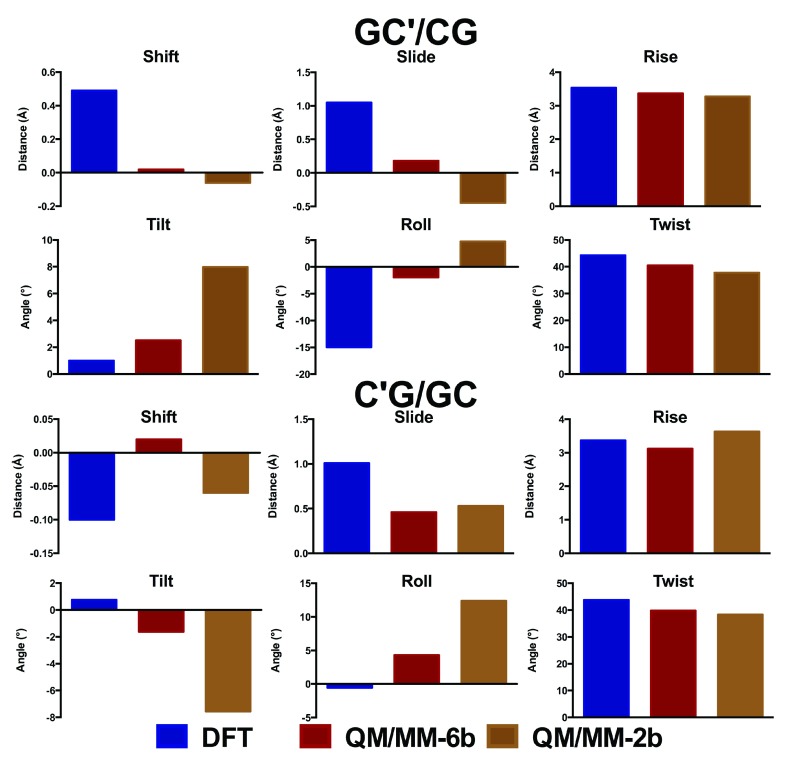
Comparison of QM/MM with DFT geometry for central GC and modifications thereof in d(GCG) (Å and °). The corresponding values are shown in Data file 3 and Data file 4.

The oligomers considered so far are close to the limit of our computational capabilities of current DFT methods (the largest structure, carboxylated d(ACA), has 962 electrons in 2743 basis functions), such that longer sequences cannot currently be routinely studied in this manner. However, they are too small to correctly represent how DNA behaves in a real system, where the conformations adopted by each base pair step depend on the neighbouring step. Moreover, simulations of nucleic acids are known to suffer problems due to greater elasticity of the terminal part of the structure (the so called “end-effect” ^41^). For these reasons, these small oligomers are inadequate models to probe the effects of epigenetic modifications on the structure of DNA. We therefore turn to hybrid QM/MM methods, in which a subset of the atoms in the system is treated with DFT, and the remainder of the system with much faster molecular mechanics methods. In order to test the validity of this approach, methylated GCG was optimised using either only two or six bases in the QM region ( Figure 4). These tests show that including only two bases in the QM region leads to significant differences in geometry to that obtained from DFT, particularly in the stagger and buckle coordinates. In contrast, including six bases in the QM region reproduces the DFT structure reasonably well. Similar observations were made from analogous treatment of methylated ACA (data not shown).

**Figure 5.**
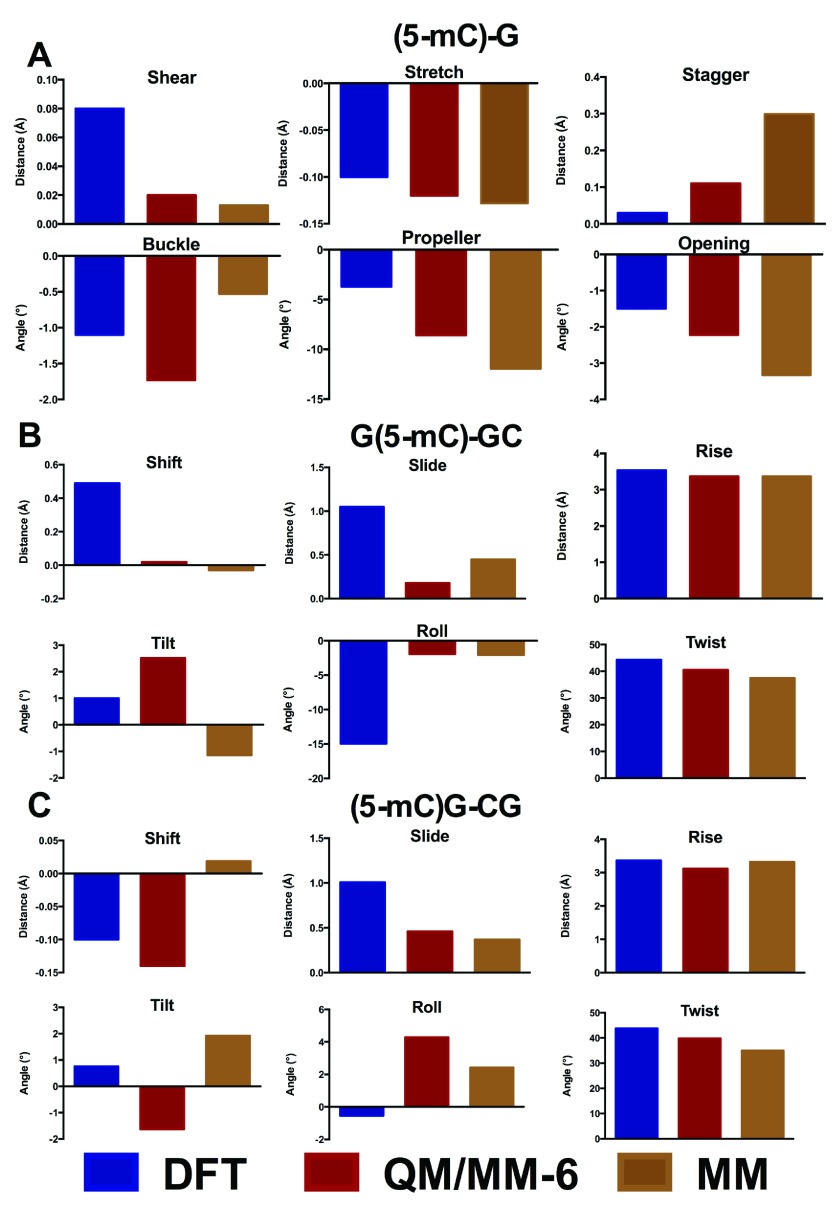
Comparison of DFT, QM/MM and molecular mechanics energy minimised (EM) geometries of d(GC´G) (Å and °). The corresponding values are shown in Data file 3– Data file 6.

As a further test, we also compared DFT and QM/MM derived structures with those optimised using the force field parameters developed in our previous work. Figure 5 shows the base-pair parameter values of the methylated structure d(GC´G) for the different methods. The MM structures provide very close values to those obtained by both QM/MM and DFT approaches, showing slight difference only in the stagger and propeller angle. We can therefore conclude that for small DNA oligomers, DFT, QM/MM and MM methods can all produce almost equally adequate DNA structures, but that QM/MM and MM approaches are more similar to one another than those obtained from DFT alone.

**Figure 6.**
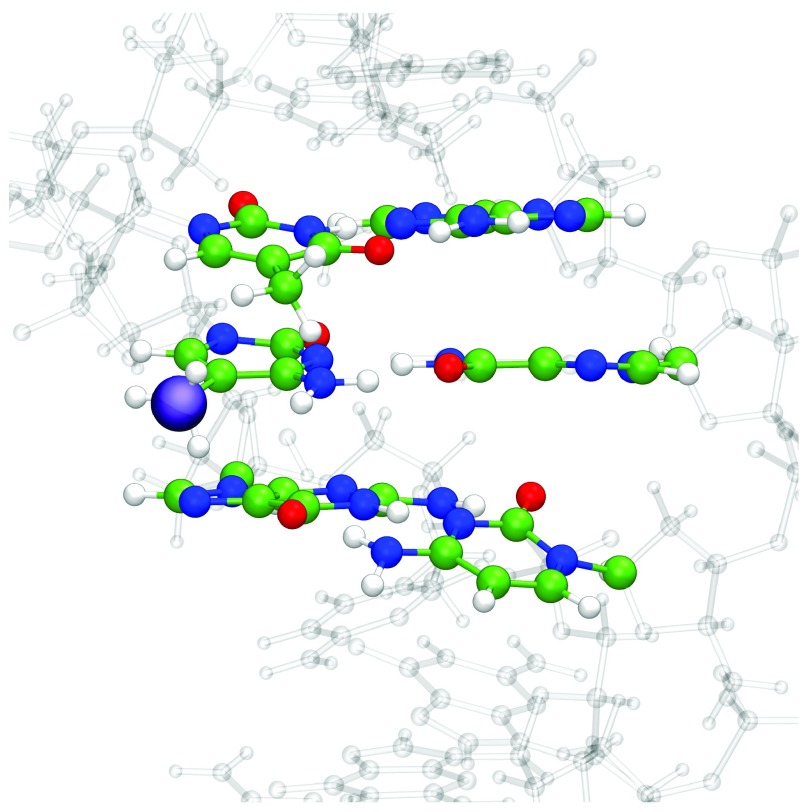
QM/MM optimised structure of 5´-ATTCGCG-3´ with 5-mC in central position, and the bases defined as QM atoms shown in CPK. A purple sphere highlights the methylation position, and water molecules and counterions have been omitted for clarity.

**Figure 7.**
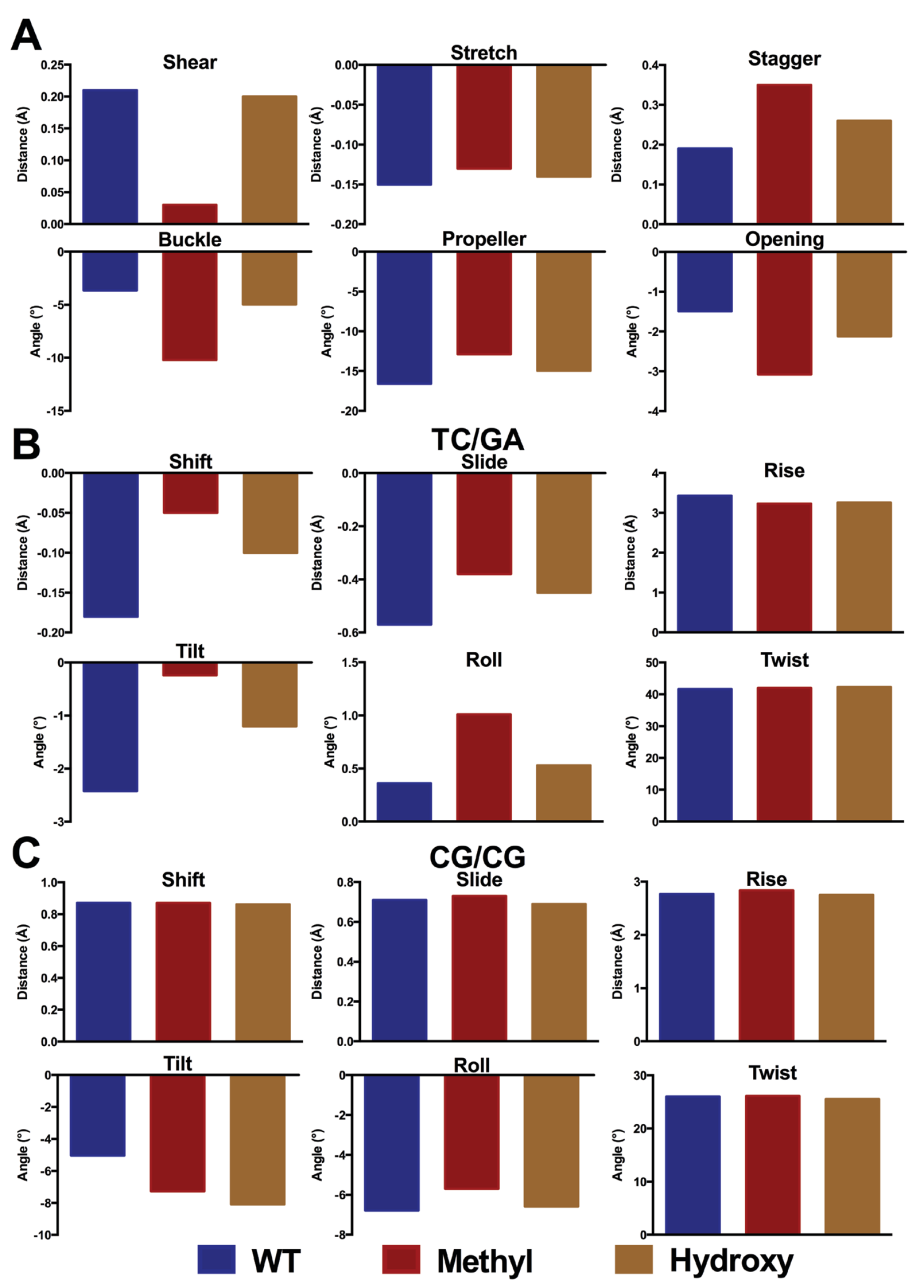
Base pair step parameters for central GC and modifications thereof in 5´-ATT CGCG-3´ (Å and °) taken from QM/MM optimisation. The corresponding values are shown in Data file 2.

QM/MM geometry optimisation with six bases in the QM region was then applied to a set of larger DNA sequences. The experimental structure of Renčiuk *et al.* ^4^ obtained using X-ray diffraction (PDB Entry 4GLG) was truncated to a sequence of 7 base pairs, i.e. 5´-ATT CGCG-3´, and the central 6 bases (TCG//CGA) assigned as QM atoms. The remaining atoms, including crystallographic water molecules and counterions, were assigned to the MM layer, and the entire system was geometry optimised. The resulting optimised structure of the system with methylated C in the central position is shown in Figure 6. Base pair and base pair step geometries of wild type, methylated, hydroxymethylated structures optimised with QM/MM, along with experimental values for methylated C, are shown in Figure 7.

We find that the structural effect of methylation is larger in this longer sequence than in the trimers considered above. Particularly, the optimised values of shear, stagger and buckle of the central base pair differ markedly between the methylated and WT forms of DNA. In contrast, the base pair step parameters exhibit rather smaller changes. For the hydroxymethylated structures, we observe similar profiles to the methylated structures. Furthermore, our simulations also allow us to probe the preferred orientation of the hydroxymethyl group: our DFT calculations predict a slight preference for the OH group to point in 3´ over 5´ and an optimised of C6-C5-C5A-O5 torsion angle of 118.4°, while previous MD simulations show this torsion to vary between 85 and 120° over 100 ns of simulation ^19^. This is in good agreement with the experimental and theoretical results of Renčiuk *et al.* ^42^, who reported values between 72 and 133° using X-ray diffraction methods.

Data of theoretical modelling of epigenetically modified DNA sequences Data file 1 Local base pair parameters for the central GC in d(GCG) and d(ACA) modifications. The shear, stretch and stagger parameters are measured in Å, and the buckle, propeller and opening parameters are measured in ˚.
Data file 2 Base pair step and local helical parameters for the central GC in d(GCG) and d(ACA) modifications. The slide and X-displacement parameters are measured in Å, and the roll, twist and inclination parameters are measured in ˚.
Data file 3 Comparison between the obtained base pair parameters for the d(GCG) using different levels of theory (DFT and the QM/MM with 2bps or 6bps in the high level layer). The shear, stretch and stagger parameters are measured in Å, and the buckle, propeller and opening parameters are measured in ˚.
Data file 4 Comparison between the obtained base step parameters for the d(GCG) using different levels of theory (DFT and the QM/MM with 2bps or 6bps in the high level layer). The slide, shift and rise parameters are measured in Å, and the tilt, roll and twist parameters are measured in ˚.
Data file 5 Values of local base pair parameters of central GC pair of d(GCG) obtained after energy minimization. The shear, stretch and stagger parameters are measured in Å, and the buckle, propeller and opening parameters are measured in ˚.
Data file 6 Values of base pair step parameters of d(GCG), obtained after energy minimization. The shift, slide and rise parameters are measured in Å, and the tilt, roll and twist parameters are measured in °.
Data file 7 Cartesian coordinates of key species. Click here for additional data file. Copyright: © 2015 Carvalho ATP et al. 2015 Data associated with the article are available under the terms of the Creative Commons Zero "No rights reserved" data waiver (CC0 1.0 Public domain dedication).

## Conclusions

Through use of modern, dispersion-corrected DFT and hybrid QM/MM methods, we have examined the structural consequences of epigenetic modifications of DNA. Concentrating on methylation and related modifications of cytosine, we show that the overall Watson-Crick base-pairing is retained, with rather small changes to hydrogen bond and stacking geometries. Despite this, some modifications have a substantial effect on the strength of intermolecular interactions: hydroxymethyl and formyl groups reduce H-bonding strength, while carboxylate increases this markedly.

Situating these modifications within the double-stranded DNA trimers GCG and ACA allows us to examine the effects on the central CG base pair and base pair steps. Base pair geometries undergo rather larger changes within ACA than in GCG, with changes in buckle and propeller angles particularly apparent. Changes to base pair steps are smaller, although some changes in shift and slide values due to modifications are evident. Optimised geometries also act as a useful test of hybrid QM/MM methods. These can reproduce DFT structures if all six bases are included in the QM region, but if only the central base pair is treated with QM significant differences result. This approach is then applied to heptamers derived from a recent X-ray crystallography; here again, the central base pair is found to be significantly disrupted, whereas base pair step parameters are largely retained.

The studies reported here deal solely with static structures, but it is well-known that DNA is a flexible system that is in constant motion at biologically relevant temperatures. In previous work, we showed that long timescale molecular dynamics was able to highlight subtle differences in structure, flexibility and solvation resulting from incorporation of 5-mC and 5-hC in several different DNA sequences. The work reported here gives new insight into the intrinsic effects of epigenetic modification of cytosine, complementing our previous molecular dynamics study ^19^ as well as providing support for the molecular mechanics force field chosen for that work.

## Data availability

The data referenced by this article are under copyright with the following copyright statement: Copyright: © 2015 Carvalho ATP et al.

Data associated with the article are available under the terms of the Creative Commons Zero "No rights reserved" data waiver (CC0 1.0 Public domain dedication). 

*Figshare:* Data of theoretical modelling of epigenetically modified DNA sequences. Doi: 10.6084/m9.figshare.1310448 ^43^

## Figures and Tables

**Figure S1.**
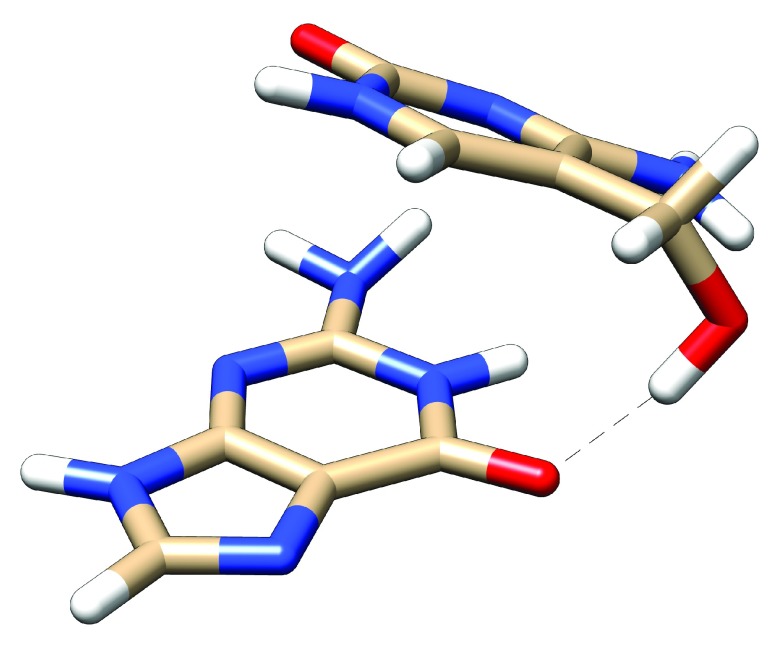
Optimised geometry of stacked 5-hC…G, showing the hydrogen bond to the O6 atom of G. Cartesian coordinates of all optimised structures from this work are provided in the data widget.
