# Supplementary material for: Theoretical modelling of epigenetically modified DNA sequences
Source: F1000Res. 2015 May 6;4:52. Originally published 2015 Feb 24. [Version 2] doi: 10.12688/f1000research.6148.2 (PMC4582758; doi:10.12688/f1000research.6148.2)
Supplement: Peer review history

# Peer review: Referee response for version 2

**DOI:** 10.5256/f1000research.6909.r8570

**Published:** 2015-09-16

**Authors:** Katja Petzold

**Affiliations:** 1 Department of Medical Biochemistry and Biophysics (MBB-Molecular Structural Biology), Karolinska Institute, Stockholm, Sweden

Thank you for the detailed answers and adjustment.

I have read this submission. I believe that I have an appropriate level of expertise to confirm that it is of an acceptable scientific standard.

# Peer review: Referee response for version 1

**DOI:** 10.5256/f1000research.6589.r8191

**Published:** 2015-04-17

**Authors:** Katja Petzold

**Affiliations:** 1 Department of Medical Biochemistry and Biophysics (MBB-Molecular Structural Biology), Karolinska Institute, Stockholm, Sweden

This paper presents a study of different simulation methods to investigate different modification of dCMp in base pairs or dsDNA and their influence of the surrounding structure.

 

1. Disparate statement in the abstract – please modify/clarify: “The same modifications within double-stranded GCG and ACA trimers exhibit rather larger structural effects” versus “but these leave the overall helical shape of DNA essentially intact.” larger changes but DNA shape of helix intact?
2. Please clarify statement in introduction: “Fascinatingly, such modifications may be heritable despite not affecting DNA sequence, although the mechanism(s) by which this could be achieved are currently unknown.” In respect to enzymes (e.g. DNA methyltransferase) known to transfer methylation from parent to daughter strands?
3. Please enhance figures for clarity.
  A: Fig 1: numbering of atoms, full name of modifications, example GC WC base pair geometry and info on parameters “role, rise twist etc…”.
  B: Fig. 2-5 & 7: please keep coherent direction of sequences e.g. GC/GC (5’-3’/5’-3’) in Fig. 2 vs Fig. 4 GC/CG, or coherent naming of modification: Fig. 2 – no indication which nucleotide is modified, Fig. 4: C’, Fig. 5: (5-mC), if mis-understood – please clarify.
  C: Please give more detail in each of the Figure caption (e.g. construct, reference structure – Fig. 7).
  D: Fig. 7: what is the reference “along with experimental values for methylated C” – are the values shown here the X-ray structure values or the X-ray Structure values optimized with QM/MM for the WT? – if it is the optimized data, than I suggest to add the experimental data uncorrected as well.
4. It is difficult to estimate the significance of the changes in structural parameters between different cytosine modifications or different simulation methods, as there are no errors/standard deviations are presented. I would suggest using a set of X-ray/NMR structures with the same sequence and/or modifications to create a standard deviation for the different parameters to give the analysis more significance (than I can estimate if a difference of 0.03Å is of importance or not: “Formyl has a larger effect overall, lengthening N3…H1-N1 and O2…H2-N2 H-bonds and hence reducing binding by over 3 kcal/mol.” Difference in h-bond length from wtC is 0.025Å and 0.049Å, respectively – seems very small, but if all GC wc bp are within of 0.01Å distance, this would be significant).
  Important for: Table 1&2 – as well important for Fig. 2-5 & 7, please adjust.
5. Describe structural/distortion findings in structure/sketches. E.g.: “largely due to a strong H-bond between the H—O of hydroxymethyl and O6 of guanine (H…O = 1.770 Å)” for a better understanding of how the structures are supposed to look like.
6. Formality: p5 first sentence “Following Zubatiuk *et al*.37,” should be “Following **Olson** *et al*.37,” OR “Following Zubatiuk *et al*. **14**,”
7. More information on the Methods and Materials would be appreciated: E.g. How extensive where the simulations/optimizations? What were the energy cutoffs?... 

I have read this submission. I believe that I have an appropriate level of expertise to confirm that it is of an acceptable scientific standard, however I have significant reservations, as outlined above.

# Response

**Published:** 2015-04-23

**Authors:** Lynn Kamerlin

**Affiliations:** Department of Cell and Molecular Biology, Uppsala University, Sweden

We thank the referee for the time taken to review our manuscript. Please find a point-by-point response below, with our responses italicised. 

1. Disparate statement in the abstract – please modify/clarify: “The same modifications within double-stranded GCG and ACA trimers exhibit rather larger structural effects” versus “but these leave the overall helical shape of DNA essentially intact.” larger changes but DNA shape of helix intact? 
*We do not see these statements as contradictory: we show that there are indeed substantial changes in H-bonding and stacking interactions, but that these are not sufficient to disrupt the overall helical structure. We have, however, now explicitly included this in the abstract to prevent reader confusion.*
2. Please clarify statement in introduction: “Fascinatingly, such modifications may be heritable despite not affecting DNA sequence, although the mechanism(s) by which this could be achieved are currently unknown.” In respect to enzymes (e.g. DNA methyltransferase) known to transfer methylation from parent to daughter strands? 
*This is certainly one key mechanism, but this is not the place to discuss in detail the biology of epigenetics, which is covered at length in references cited. We have made this point more explicit in the introduction and refer the reader to reference 1 for further information about currently proposed mechanisms.*
3. Please enhance figures for clarity. 
*The figures have been modified as outlined below and we hope the improved version is now clearer to the reader.*
  A: Fig 1: numbering of atoms, full name of modifications, example GC WC base pair geometry and info on parameters “role, rise twist etc…”.
*Numbering has been added to Figure 1, as has a representation of CG base pair. Roll, rise, twist *etc. *are widely used in DNA studies and should not need re-definition here.*
  B: Fig. 2-5 & 7: please keep coherent direction of sequences e.g. GC/GC (5’-3’/5’-3’) in Fig. 2 vs Fig. 4 GC/CG, or coherent naming of modification: Fig. 2 – no indication which nucleotide is modified, Fig. 4: C’, Fig. 5: (5-mC), if mis-understood – please clarify.
*The legend for figures 2 to 4 has been altered to explain that central C has been modified.*
  C: Please give more detail in each of the Figure caption (e.g. construct, reference structure – Fig. 7).
*Legend for Figure 7 has been expanded to clarify source of data.*
  D: Fig. 7: what is the reference “along with experimental values for methylated C” – are the values shown here the X-ray structure values or the X-ray Structure values optimized with QM/MM for the WT? – if it is the optimized data, than I suggest to add the experimental data uncorrected as well.
*This was an oversight from a previous draft: Figure 7 does not contain experimental data, and this has been removed from the manuscript. Inclusion of further data from experiment would make this figure too cluttered and difficult to read.*
4. It is difficult to estimate the significance of the changes in structural parameters between different cytosine modifications or different simulation methods, as there are no errors/standard deviations are presented. I would suggest using a set of X-ray/NMR structures with the same sequence and/or modifications to create a standard deviation for the different parameters to give the analysis more significance (than I can estimate if a difference of 0.03Å is of importance or not: “Formyl has a larger effect overall, lengthening N3…H1-N1 and O2…H2-N2 H-bonds and hence reducing binding by over 3 kcal/mol.” Difference in h-bond length from wtC is 0.025Å and 0.049Å, respectively – seems very small, but if all GC wc bp are within of 0.01Å distance, this would be significant). 
*It is indeed difficult to estimate the significance of changes in geometry: these static DFT and QM/MM calculations do not yield standard deviations. It would indeed be interesting to extract experimental information to estimate variability across structures, but this would be a whole new project, and is therefore out of the scope of the present work.*
  Important for: Table 1&2 – as well important for Fig. 2-5 & 7, please adjust.
*As outlined above, we do not have suitable data with which to adjust these tables and figures.*
5.  Describe structural/distortion findings in structure/sketches. E.g.: “largely due to a strong H-bond between the H—O of hydroxymethyl and O6 of guanine (H…O = 1.770 Å)” for a better understanding of how the structures are supposed to look like. 
*We have added a figure for this structure to supporting information, and stress that all optimised coordinates have been deposited should readers wish to assess further detail.*
6. Formality: p5 first sentence “Following Zubatiuk *et al*.37,” should be “Following **Olson** *et al*.37,” OR “Following Zubatiuk *et al*. **14**,”
*We thank the referee for spotting this error, and have corrected it to Following Zubatiuk et al.14,”*
7. More information on the Methods and Materials would be appreciated: E.g. How extensive where the simulations/optimizations? What were the energy cutoffs?...  
*All DFT and QM/MM calculations used Gaussian09 default convergence criteria for SCF calculation and geometry optimisation: a statement to this effect has been added to the methods section. Details of MM calculations are identical to those from our previous work (ref 19): again, a statement has been added to this effect.*

# Peer review: Referee response for version 1

**DOI:** 10.5256/f1000research.6589.r7803

**Published:** 2015-03-27

**Authors:** Célia Fonseca Guerra

**Affiliations:** 1 Department of Theoretical Chemistry and Amsterdam Center for Multiscale Modeling, VU University Amsterdam, Amsterdam, Netherlands

This paper presents an interesting theoretical studty on epigenetically modified DNA.

Legend of  Figure 1: Please include the numbering, so that non-experts can follow the rest of the text.

Page 4: “The presence of a hydroxymethyl slightly weakens the N4-H4...O6“ Is there an internal hydrogen bond that is competing with the N4-H4•••O6 hydrogen bond? Please explain.

Page 4 “Formyl has a larger effect overall, lengthening N3...H1-N1 and O2...H2-N2 H-bonds and hence reducing binding by over 3 kcal/mol. ” This can easily be understood because N3 and O2 become less negative due to the electron withdrawing effect. See *Chem. Eur. J.***2006**, *12*: 3032-3042, *Chem. Eur. J.***1999**, *5:* 3581-3594 and *Chem. Eur. J.*** 2011**, *17*: 8816-8818 and use these publications to explain these effects on the hydrogen bonds. The epigenetic modifications can be considered to be substituent effects and therefore the changes in the hydrogen bonds can be easily explained.

Table 1: What are the hydrogen bonds lengths meant here? N4•••O6 or H4•••O6. The preference would be N4•••O6.

I have read this submission. I believe that I have an appropriate level of expertise to confirm that it is of an acceptable scientific standard.

# Response

**Published:** 2015-04-23

**Authors:** Lynn Kamerlin

**Affiliations:** Department of Cell and Molecular Biology, Uppsala University, Sweden

We again thank the reviewer for the time taken to referee the manuscript. Please see our point-by-point response below.

- Legend of Figure 1: Please include the numbering, so that non-experts can follow the rest of the text.
*Numbering has been added to Figure 1.*
- Page 4: “The presence of a hydroxymethyl slightly weakens the N4-H4...O6“ Is there an internal hydrogen bond that is competing with the N4-H4•••O6 hydrogen bond? Please explain.
*The OH group of hydroxymethyl is found to lie close to H4, but the lengths reported in Table 1 put this “contact” outside typical ranges of N-H…O hydrogen bonds, such that we prefer not to refer to a hydrogen bond, but rather the proximity of groups.*
- Page 4 “Formyl has a larger effect overall, lengthening N3...H1-N1 and O2...H2-N2 H-bonds and hence reducing binding by over 3 kcal/mol. ” This can easily be understood because N3 and O2 become less negative due to the electron withdrawing effect. See Chem. Eur. J. **2006**, 12: 3032-3042, Chem. Eur. J. **1999**, 5: 3581-3594 and Chem. Eur. J. **2011**, 17: 8816-8818 and use these publications to explain these effects on the hydrogen bonds. The epigenetic modifications can be considered to be substituent effects and therefore the changes in the hydrogen bonds can be easily explained.
*We completely agree that these trends can be understood as substituent effects, and have therefore added both text to reflect this and the suggested references to the relevant section of the Results and Discussion.*
- Table 1: What are the hydrogen bonds lengths meant here? N4•••O6 or H4•••O6. The preference would be N4•••O6.
*H-bond lengths are reported as H…Y, since the alternative depends on angular geometry of the X-H…Y system. In any case, full coordinates have been deposited as Supporting Information in case interested parties wish to extract X…Y distances.*
